# Gut microbiota and metabolites in estrus cycle and their changes in a menopausal transition rat model with typical neuroendocrine aging

**DOI:** 10.3389/fendo.2023.1282694

**Published:** 2023-12-15

**Authors:** Ruoxi Dai, Jianqin Huang, Liyuan Cui, Ruiqi Sun, Xuemin Qiu, Yan Wang, Yan Sun

**Affiliations:** ^1^ Hospital and Institute of Obstetrics and Gynecology, Fudan University, Shanghai, China; ^2^ The Academy of Integrative Medicine, Fudan University, Shanghai, China; ^3^ Shanghai Key Laboratory of Female Reproductive Endocrine-Related Disease, Hospital of Obstetrics and Gynecology, Fudan University, Shanghai, China; ^4^ Department of Clinical Medicine, Clinical College of Anhui Medical University, Hefei, China

**Keywords:** gut microbiota, metabolites, hypothalamus, luteinizing hormone, aging

## Abstract

**Background:**

Neuroendocrine alterations in the mid-life hypothalamus coupled with reproductive decline herald the initiation of menopausal transition. The certain feature and contribution of gut microflora and metabolites to neuroendocrine changes in the menopausal transition remain largely unknown.

**Methods:**

Fecal samples of rats experiencing different reproductive stages were collected and processed for 16S rRNA and liquid chromatography–mass spectrometry sequencing. The differences of gut microbiota and metabolites between young and middle-aged rats during proestrus and diestrus were analyzed, and their relationships to neuroendocrine aging were then examined.

**Results:**

At the genus level, *Anaeroyorax, Rikenella, Tyzzerella_3, and Atopostipes* were abundant at proestrus, while *Romboutsia, Turicibacter, Clostridium_sensu_stricto_1, Ruminococcaceae_NK4A214_group, CHKCI002, Ruminococcaceae_UCG-010, Staphylococcus, Family_XII_AD3011_group, Ruminococcaceae UCG-011, and Christensenellaceae_R_7_group* were enriched in the diestrus of middle-aged rats. *DNF00809, Phocea, and Lachnospiraceae_UCG-006* were found abundant during proestrus instead, while *Bacteroides, Lactobacillus, Erysipelatoclostridium, Anaeroplasma, Anaerofustis, Parasutterella, and Enterococcus* were enriched at the diestrus of young female individuals. Discriminatory metabolites were identified involving 90 metabolic pathways among the animal sets, which were enriched for steroid hormone biosynthesis, arachidonic metabolism, primary bile acid synthesis, and ovarian steroidogenesis. A total of 21 metabolites lacking in hormone-associated changes in middle-aged female individuals presented positive or negative correlations with the circulating luteinizing hormone, bile acid, fibroblast growth factor 19, and gut hormones. Moreover, close correlations were detected between the intestinal bacteria and their metabolites.

**Conclusion:**

This study documents specific gut microbial composition changes and concomitant shifting trends of metabolites during menopausal transition, which may initiate the gut–brain dysfunction in neuroendocrine aging.

## 1 Introduction

Age-related alterations at the hypothalamus have been documented and undoubtedly contribute to the cessation of reproductive function that leads to menopause (1). Before the evident emergence of irregular menstruation, numerous neurochemicals (i.e., glutamate, gamma aminobutyric acid (GABA), norepinephrine (NE), and kisspeptin) required for the preovulatory gonadotropin-releasing hormone (GnRH) release appear to be dampened during middle age (2, 3), manifested by reduced neuronal activation in the rostral periventricular third ventricle (RP3V) and an attenuation of GnRH/luteinizing hormone (LH) surge (4). Given that a specific area of the hypothalamus is the interface between the brain and peripheral endocrine systems, environmental factors may potentially program the age-associated hypothalamic defects. As suggested by preclinical studies, intestinal microflora and metabolites are probably involved in neurogenesis and central biochemistry as critical environmental factors (5–7). Altered interactions of the gut–brain microbiota are inclined to cause well-known gut–brain disorders bidirectionally (8, 9) and prone to mediate the pathogenesis of several brain diseases encompassing Parkinson’s disease and emotional disorders (10, 11). It is, however, not clearly elucidated how the community of gut microbe changes in menopausal transition and if those changes are causally linked to neuroendocrine defects.

The gut microbiota, an accumulation of intestinal microorganisms, exerts impacts on the endocrine system *via* microbial metabolites by activating the enteric nervous system (12). The gut microbe-produced metabolites can play either excitatory or inhibitory roles through various mechanisms, such as accelerating anabolic metabolism by providing supportive metabolic flux (13), serving as inhibitors of competitive enzymes (14), or embellishing signal proteins (15, 16). The abundance and composition of the host microbial community shift with estrous cycles (17). Santos-Marcos JA et al. explored the disparities in the intestinal microflora of women experiencing premenopause and post-menopause and found that the *Firmzicutes*/*Bacteroides* ratios and the richness of *Lachnospira* and *Roseburia* were comparatively higher, but the abundances of *Prevotella*, *Parabacteroides*, and *Bilophila* relatively dropped in postmenopausal women (18). In polycystic ovary syndrome (PCOS) patients, while *Parabacteroides distasonis* and *Bacteroides fragilis* producing GABA increased, *Escherichia coli* increases paralleled with serum LH/follicle-stimulating hormone (FSH) ratio as well (19). Some important factors have been declared to associate the intestinal microbiota with the hypothalamus, that is, microbial metabolites (20) and neurotransmitters (21), including dopamine, NE, serotonin, or GABA, which are vital for the regulation of gonadotropin release and sustainment of energy balance. However, limited evidence pertaining to shifting microecology or its derived metabolic products has been found in female individuals experiencing menopausal transition with hypothalamus aging.

Rodents are devoted to models simulating the reproductive physiology of female individuals because they bear a striking similarity to human beings in genetics and physiology. The cycle patterns, serum LH, FSH, estradiol (E2), and progesterone (P) levels during the rodents’ estrous cycle share similarities with the human menstrual cycle (22). The current study aims to examine features of gut microbiome and metabolites between middle-aged (MA) and young (Y) rats at diestrus and proestrus and to analyze the associations of gut-related metabolites with hypothalamic alterations during menopausal transition. This study will deepen our understanding of the neuroendocrine alterations during menopausal transition in terms of gut microenvironments and metabolites.

## 2 Materials and methods

### 2.1 Animal care

Young (Y; 2- to 3-month-old) and middle-aged (MA; 9- to 10-month-old, retired breeders) female rats (Sprague Dawley, Charles River, Beijing) were equally fed with radiation-sterilized lab rodent feed (number 1010086; Jiangsu Xietong Pharmaceutical Bio-engineering Co., Ltd.) and water *ad libitum*. A 12-h light/12-h dark cycle (lights on at 8 a.m.) was held. All experimental schemes realized on the animals were authorized by the Institutional Animal Care and Use Committee of Fudan University.

### 2.2 Estrous cycle determination and fecal collection

Estrous periodicity was detected by vaginal smear for at least consecutive two cycles (10 days) (**Supplementary Figure S3**). Only rats in possession of two regular estrous periods (4 to 5 days) were included for the subsequent assays (23–26). On the basis of age and estrous stage, the rats were assigned to one of the following subgroups respectively and individually: young, proestrus and diestrus (Y-P and Y-D) and middle-aged, proestrus and diestrus (MA-P and MA-D). All fecal specimens were harvested and quickly chilled in liquid nitrogen and finally transferred to -80°C in a refrigerator for subsequent experiments.

### 2.3 Genomic DNA obtaining and 16S rRNA gene arraying

The genomic DNA of the samples was secured by using a fecal DNA extraction kit (Beijing Tiangen Biochemical Technology Corporation Ltd.). After determining the purities and densities of the total DNA by electrophoresis, a proper volume of the sample was transferred to a new 1.5-mL Eppendorf tube and deliquated in double-distilled water to 1 ng/μL. Then, the v3–v4 region of the 16S rRNA gene was expanded by PCR using the diluted samples as templates. The barcoded and extended primers specified in the amplification procedure were 343F (5′-TACGGRAGGCAGCAG-3′) and 798R (5′-AGGGTATCTAATCCT-3′). The products were thoroughly mingled in accordance with the concentration after the detection by electrophoresis. Then, the PCR products were refined with the help of 2% of 1X TAE gel electrophoresis to obtain the target bands. The library was created by using Ion Plus Fragment Library Kit 48 RXNS from Thermo Fisher Scientific. After having been qualified by Qubit quantitative and library testing, the library was processed in computer sequencing using Ion S5TMXL of Thermo Fisher Scientific.

### 2.4 Sequence processing and data analysis

The low-quality parts of reads were cut, and then the processed reads were screened with the species annotation database taken as a comparison in order to explore chimera sequences by Cutadapt (v1.9.1) (27). Clean Reads, the final effective data, were obtained with those chimera sequences being removed. All Clean Reads were gathered using Uparse software (Uparse v7.0.1001) (28), and the sequences were classified as operational taxonomic units (OTUs) with the default identity set at 97%. The OTU sequences were annotated by species using Mothur method (29) and the database SSUrRNA of SILVA 132 (the borderline was determined from 0.8 to 1.0) (30). The MUSCLE software (v3.8.31) was used to derive the phylogenetic relationships from the overall OTUs that were aligned by fast multisequence alignment (31). The alpha diversity and beta diversity of the bacterial species were identified by using the QIIME software (v1.9.1) (32). Unifrac distances were computed to evaluate the assembly and disparity among OTUs (33, 34), and unweighted pair group method with arithmetic mean (UPGMA) was built. The linear discriminant analysis effect size (LEfSe) was employed to analyze the discriminations of microbial abundances of each animal set (35). The functions of all the samples were predicted by using the Tax4Fun software (36).

### 2.5 Liquid chromatography–mass spectrometry analysis of fecal metabolites

The metabolites were drawn by the way of adding 20 mL separation buffer (methanol/acetonitrile/water on a scale of 2:2:1 in volume) to every 1 mg feces. A 10-mL mix of all the samples was isolated to serve as quality control (QC) and an evaluation criterion of steadiness during the research. Then, 2-mL mix per sample was extracted to obtain the metabolic signals of all the samples with liquid chromatography–mass spectrometry (LC-MS) (ACQUITY UPLC I-Class plus, Waters). The parameters intercalated in the metabolomic analyzing software Progenesis QI v2.3 to guarantee the quality of raw data are described as follows: 5 ppm precursor tolerance, 10 ppm product tolerance, and 5% product ion threshold (Nonlinear Dynamics, Newcastle, UK). The total produced data were standardized by the internal standard normalization method, and the outcomes were laid out in the shape of peak-related values, that is, the ratio of the peak areas of the test sample *versus* the internal standard sample. The Lipidmaps (V2.3), Metlin, EMDB, PMDB, and self-built databases were referred to for the characterization of compounds based upon accurate mass-to-charge ratio (*m*/*z*), secondary fragmentation, and isotopic distributions. A data matrix combining the cation and anion data was introduced into the R software for the purpose of conducting principal component analysis (PCA), which portrayed the comprehensively distributive profile of the metabolomes of the four subgroups and the steadiness of the analyzing procedures. Orthogonal partial least-squares-discriminant analysis (OPLS-DA) and PLS-DA were applied to characterize the differential metabolites among groups. The engagement of each variable in group discrimination was arranged by variable importance of projection (VIP) values attained from the OPLS-DA model. Two-tailed Student’s *t*-test was subsequently applied to validate the statistical significance of the differential metabolites across all the animal sets. Metabolites with distinctiveness were defined when VIP values > 1.0 and *P*-values < 0.05. Differential metabolic pathways were detected by MetPA (37). Moreover, the correlativity between microbiota and metabolites was assessed by using Spearman correlation analysis.

### 2.6 Immunofluorescence for c-Fos

The animals were sacrificed at around 4 pm in the proestrus afternoon (38, 39). The brain samples were collected after anesthesia followed by decapitated execution and put in 4% paraformaldehyde at 4°C for one night and dehydrated in 30% sucrose solution. Six series of coronal sections (30 µm) containing the anteroventral periventricular nucleus (AVPV) and preoptic area (POA) were assembled, with every sixth section of each series contained (24, 25). The sections were cryopreserved (40) at -20°C until the conduct of tissue immunolabeling techniques. Subsequent to the slices being flushed in potassium PBS (KPBS) [0.05 M, pH 7.4] was the immersion in rabbit anti-c-Fos (1:100, Servicebio) antibodies deliquated in 5% bovine serum albumin and the rinse in PBS. Following incubating with fluorescein isothiocyanate anti-rabbit secondary antibody, the slices were sealed by using anti-fluorescence quenching tablets.

### 2.7 Serum endocrine-related parameter assay

After the anesthesia and decapitation of rats, terminal blood sample was extracted from the trunk. The whole blood samples were rested at room temperature for 1 to 2 h and then centrifugated at 7,500 rpm for 15 min. The serum samples were transferred into clean tubes and preserved at -80°C. The levels of LH, E2, P, fibroblast growth factor (FGF)-19, ghrelin, neuropeptide Y (NPY), peptide YY (PYY), and glucagon-like peptide-1 (GLP-1) in the serum were measured using enzyme-linked immunosorbent assay, and the concentrations of bile acid were measured by using colorimetric assay, which was performed by Beijing Sino-UK Institute of Biological Technology (Chaoyang, Beijing). (The levels of FSH, E2, and P are presented in **Supplementary Figure S3**).

### 2.8 Hypothalamic dissection

MA and Y rats were rapidly decapitated after anesthesia in the proestrus afternoon aiming for RNA-sequencing procedures. Briefly as the preceding experiments (24, 25, 41), the anterior hypothalamus (encompassing POA and AVPV) was frozen quickly and preserved at -80°C for the subsequent quantification of RNA.

### 2.9 RNA extraction and transcriptomic sequencing

RNA was withdrawn from the nuclei by means of using RNeasy minikit (number 74104; Qiagen). Libraries of sequences were then organized by means of using the NEBNext Ultra RNA Library Prep kit as stated in the instructor’s recommended guidance. In total, 50 bp paired-end reads were attained from the library preparations which were arrayed by using NovaSeq 6000 (Illumina, Inc.).

### 2.10 RNA-seq analysis

The quality control and junction trimming of raw data were performed by using the Trim Galore software. The featureCounts function (42) of the Subread software (43) was employed for genomic analysis. The matrix illustrating gene expressions was standardized to transcripts per million mapped reads. The differentially expressed genes (DEGs) of the MA versus Y were sorted out by using the DESeq2 R package (*P* value: 0.05; log FC: 0.1) (44). The analysis of the enriched functions of DEGs was carried out using the Clusterprofiler R package (45).

### 2.11 Statistical analysis

The process of statistical analyses was implemented by using the GraphPad Prism software (v9.0.0) and R software (v3.3.2). Data were displayed as mean ± SEM. Student’s *t*-test was performed to detect differences in the numbers of c-Fos^+^ cells and the levels of serum hormones. *P*-values were corrected by Benjamini–Hochberg (BH) correction. The relationships among the metabolic substances or in the midst of microflora and metabolites were reckoned with the application of Pearson’s rank correlation analysis. Significant disparities were attained when *P* < 0.05. The significant threshold for the hypothesis tests was set at *α* = 0.05 and was amended by BH correction to reckon with a maximum of 5% viability (*q* = 0.05) of false positives.

## 3 Results

### 3.1 Diversity and compositional characteristics of the gut microbiota

**Figure 1:**
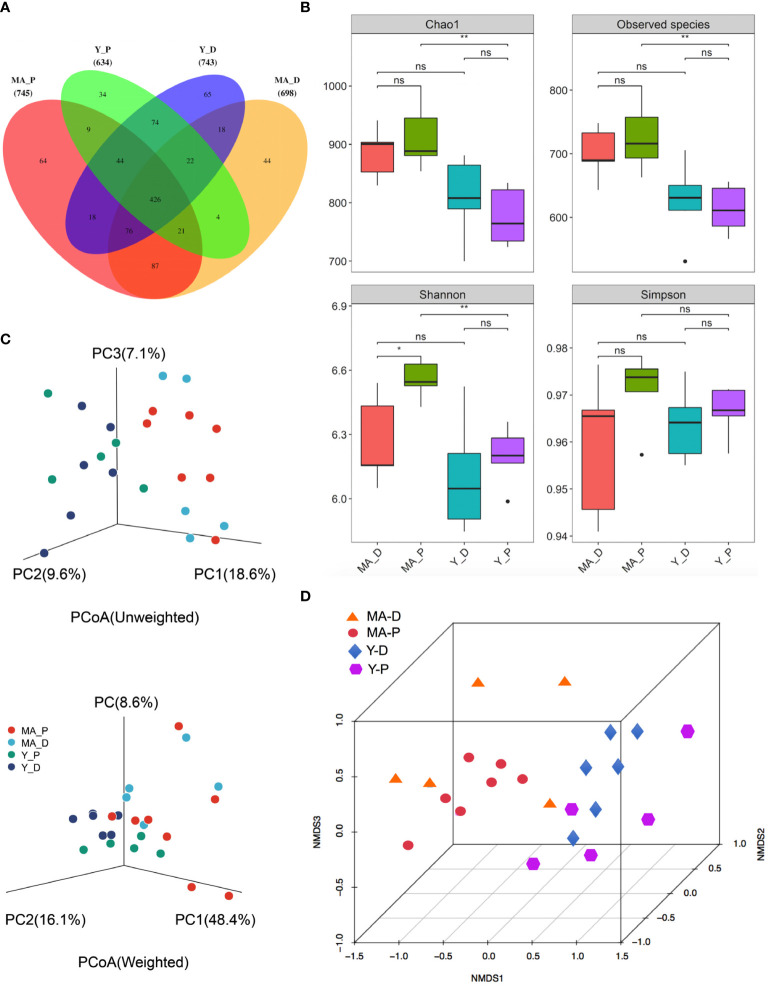
Gut bacteria diversity and cluster analysis in different groups. **(A)** Venn diagram of the gut microbiota. **(B)** The alpha diversity of the fecal microorganisms is estimated by the Chao1, observed species, Shannon, and Simpson indices. *P < 0.05, **P < 0.001; ns, not significant. **(C)** Principal coordinate analysis plots of the relative abundance. **(D)** Beta diversity analysis of the nonmetric multidimensional scaling plot of the fecal bacteria. *n* = 5–7 for each group.

A total of 1,003,162 16S rRNA reads with high qualities were totally secured, with a median read count of 35,680 (range, 26,412–38,236) per sample. As a result of categorization, 3,100 and 4,279 OTUs were acquired in the MA-D and MA-P rats, while 3,011 and 2,780 OTUs were found in Y-D and Y-P female individuals, respectively (**Supplementary Table 1**, **Supplementary Figure S1A**). The sufficiency of the sampling efforts was strongly supported by the rarefaction curves (**Supplementary Figure S1B**) of all the specimens. Based on the abundance list of OTUs, the Venn diagram depicts the OTUs both particularly in each subgroup and commonly among animal sets. There were 745 OTUs and 64 unique OTUs in MA-P rats, 698 OTUs and 44 unique OTUs in MA-D rats, 634 OTUs and 34 unique OTUs in Y-P rats, and 743 OTUs and 65 unique OTUs in Y-D rats (**Figure 1A**). To determine the variances in bacterial variety among the sets, sequences were arranged to evaluate the alpha diversity and beta diversity. The Chao1 (913.00 ± 20.57 versus 775.8 ± 22.46, *P* = 0.0025), observed species (726.3 ± 19.12 versus 613.1 ± 17.05, *P* = 0.0025), and Shannon (6.55 ± 0.03 versus 6.20 ± 0.06, *P* = 0.0025) indices presented statistical significance between the MA-P and Y-P groups (**Figure 1B**). The Shannon (6.55 ± 0.03 versus 6.27 ± 0.09, *P* = 0.0303) also showed a significant difference when the MA-D was compared to the MA-P, whereas the differences of the Simpson (0.76 ± 0.23 versus 0.85 ± 0.09, *P* = 0.0758) were not statistically significant when compared among all the sets (**Figure 1B**). Both the unweighted and weighted principal coordinate analysis (PCoA) plots separated every two sets on the strength of the first three PCoA (**Figure 1C**). The beta diversity of intestinal microflora was also analyzed by nonmetric multidimensional scaling (NMDS) (**Figure 1D**).

**Figure 2:**
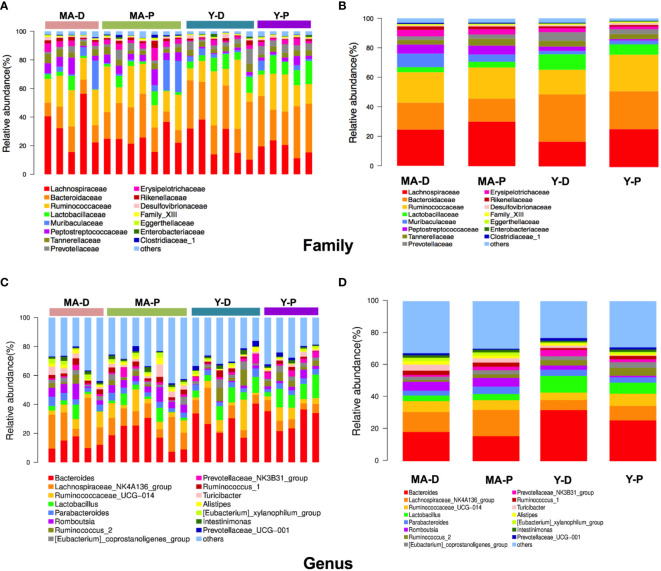
Relative abundances of distinct gut microbiota in the middle-aged and young rats at proestrus and diestrus. **(A, B)** Family level. **(C, D)** Genus level. *n* = 5–7 for each group.

Afterwards, we plotted the composition of the intestinal microbiota species in each group in cluster histograms. At the family level, the microbiomes of the four sets were dominated by *Lachnospiraceae*, *Bacteroidaceae*, and *Ruminococcaceae*. In the young animals, the abundances of *Lactobacillaceae*, *Bacteroideceae*, *Peptostreptococcaceae*, *Prevotellaceae*, and *Erysipelotrichaceae* relatively decreased, whereas that of *Lachnospiraceae* and *Ruminococcaceae* increased in the Y-P when compared with the Y-D (**Figures 2A, B**). In the middle-aged group, however, the above-mentioned changes disappeared in the MA-P in comparison with the MA-D. The relative abundances of *Muribaculaceae* showed a notable reduction in the MA-P rats in comparison to the MA-D. This family change was not observed in the young animals (**Figures 2A, B**). At the genus level, the richness of *Bacteroides*, *Lactobacillus*, *Parabacteroids*, *Romboutsia*, *Turicibacter*, and *Prevotellaceae_NK3B31_group* in the feces of Y-P were comparatively reduced, while the *Lachnospiraceae_NK4A136_group*, *Ruminococcaceae_UCG-014*, and *Ruminocococcus_1 and _2* were increased when compared with the Y-D (**Figures 2C, D**). Nevertheless, the above-mentioned changes of *Ruminococcaceae_UCG-014*, *Lactobacillus*, and *Romboutsia* were not found in the MA-P rats. Particularly, the *Prevotellaceae_NK3B31_ group* that decreased in the Y-P showed an opposite feature in the MA-P.

### 3.2 Screening of differential microbiota at proestrus and diestrus

**Figure 3:**
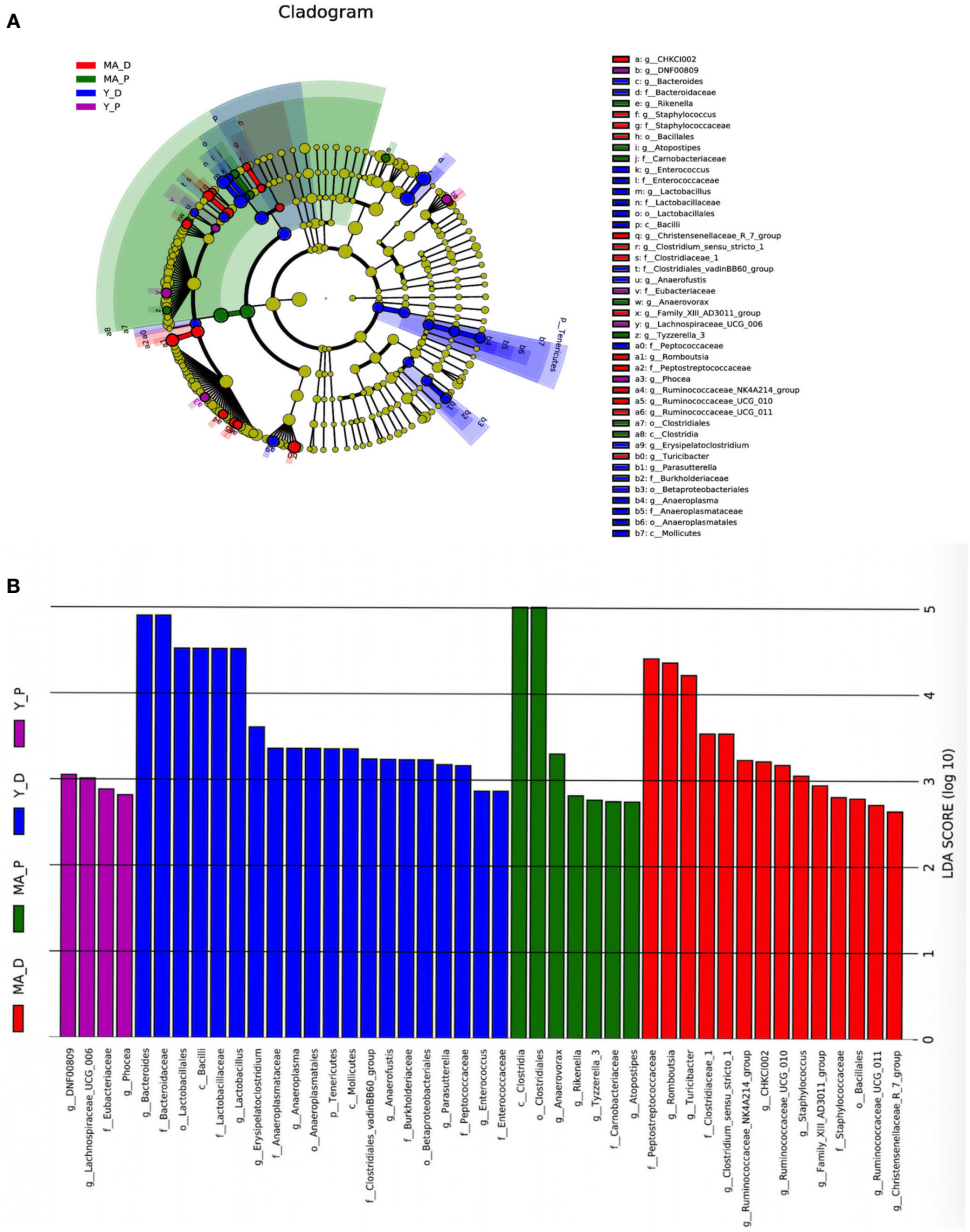
Linear discriminant analysis integrated with effect size. **(A)** Cladogram showing the distribution of microbes correlated with the middle-aged (MA) and young (Y) groups. **(B)** The differences in abundance in the MA and Y groups at proestrus and diestrus. *n* = 5–7 for each group.

Taken into consideration that this discriminating analysis could not secern the principal taxon, LEfSe was used to make out the specific microbe in different groups as presented by a cladogram (**Figure 3A**). The particularly enriched microorganisms at the genus level containing, in the middle-aged group, *Romboutsia*, *Turicibacter*, *Clostridiaceae_1*, *Clostridium_sensu_stricto_1*, *Ruminococcaceae_NK4A214_group*, *CHKCI002*, *Ruminococcaceae_UCG-010*, *Staphylococcus*, *Family_XIII_AD3011_group*, *Ruminococcaceae_UCG-011*, and *Christensenellaceae_R_7_group* were the most abundant microbiota in the MA-D, but *Anaerovorax*, *Rikenella*, *Tyzzerella_3*, and *Atopostipes* were enriched in the MA-P (**Figures 3A, B**). In the young animals, several opportunistic pathogens including *Bacteroides*, *Lactobacillus*, *Erysipelatoclostridium*, *Anaerofustis*, *Parasutterella*, and *Enterococcus* were substantially overrepresented (linear discriminant analysis (LDA) scores (log10) > 2.8) in the Y-D, whereas *DNF00809*, *Lachnospiraceae UCG-006*, and *Phocea* were more abundant in the Y-P-enriched bacteria at the family and other levels (see **Figures 3A, B**).

### 3.3 Functional enrichment analysis of microbiota

**Figure 4:**
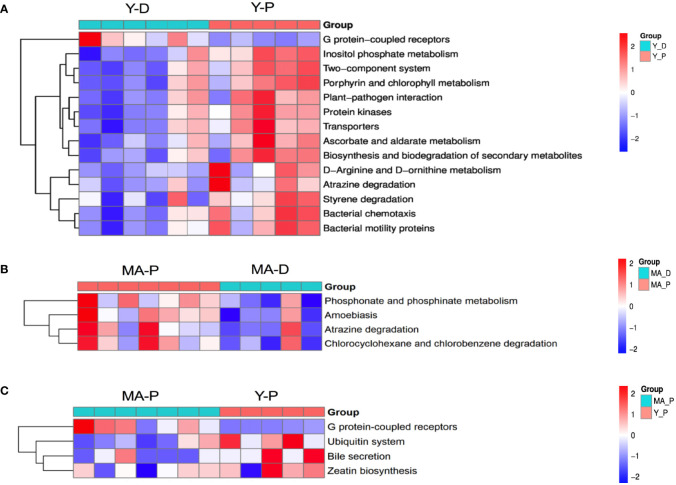
Functional characterization of gut microbes. Based on the functional classifications of the Kyoto Encyclopedia of Genes and Genomes database, the functional categories between groups were compared. Difference between **(A)** Y-D and Y-P, **(B)** MA-P and MA-D, and **(C)** MA-P and Y-P. *n* = 5–7 for each group.

With PICRUSt prediction and Kyoto Encyclopedia of Genes and Genomes (KEGG) annotation, we found that, at KEGG level 3, the Y-P showed high activity in G protein-coupled receptors, inositol phosphate metabolism, two-component system and porphyrin and chlorophyl II metabolism, and so on when compared with the Y-D (**Figure 4A**), whereas the MA-P showed high activity in phosphonate and phosphinate metabolism, amoebiasis, atrazine degradation, and chlorocyclohexane and chlorobenzene degradation when compared with the MA-D (**Figure 4B**). Unlike the Y-P group, the MA-P group showed significant differences in G protein-coupled receptors, ubiquitin system, bile secretion, and zeatin biosynthesis (**Figure 4C**).

### 3.4 Nontargeted fecal metabolomics analysis

We subsequently analyzed the metabolomics of fecal samples solely by performing a nontargeted LC–MS-based metabolomics approach. The PCA of fecal samples displayed a clear separation in metabolites of the middle-aged rats from the young, which revealed that fecal metabolome was regulated by age. The PCA also displayed a separation between the Y-P and Y-D groups, which revealed that fecal metabolome was also regulated by hormones in the young rats. However, such a separation was not identified between the MA-P and MA-D groups (**Supplementary Figure S2A**).

**Figure 5:**
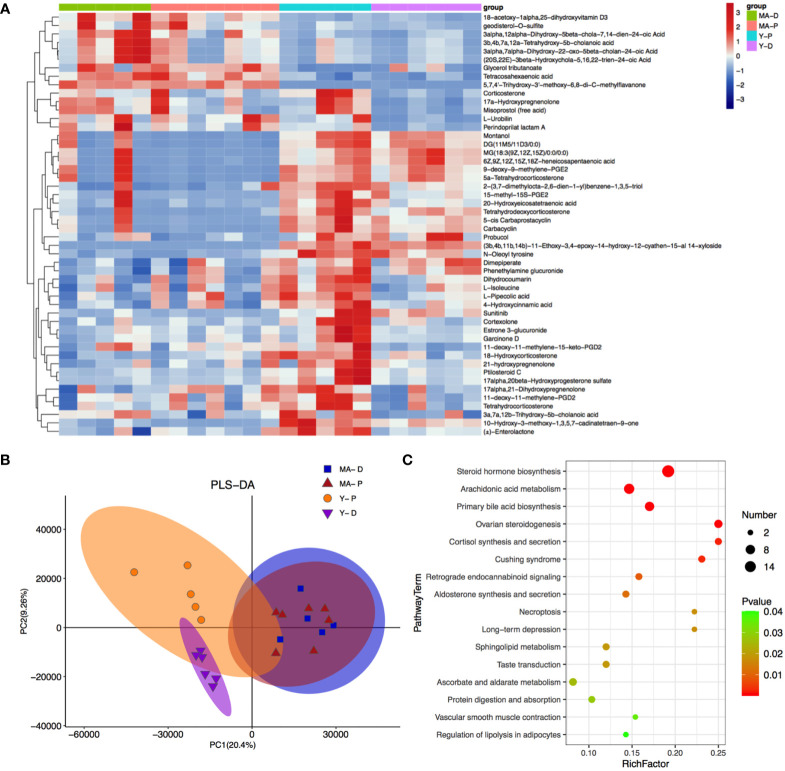
Fecal metabolites and associated enriched pathways. **(A)** Heat map showing the differential metabolites among the four groups. **(B)** Partial least-squares-discriminant analysis displaying the grouped discrimination of the middle-aged and young groups. **(C)** Kyoto Encyclopedia of Genes and Genomes enriched pathways of important metabolites. *n* = 5–7 for each group.

A total of 4,265 and 7,402 metabolites were excavated from the feces. The PLS-DA R2 values verge on 1 (**Figure 5B**). The borderline was established as VIP value > 1.0 and fold change (FC) > 2.0 or FC < 0.5 and *P* < 0.05 (**Supplementary Figure S2B**), and the metabolisms with distinct differences were sifted through as shown above (**Figure 5A**). The featured metabolites in the MA and Y groups are gathered at proestrus and diestrus, respectively, indicating that MA and Y rats at proestrus and diestrus modulate metabolites in different patterns. There were totally 777 distinctly different metabolites in the MA and Y groups, among which 277 metabolites were notably changed between the Y-P and Y-D groups, while 90 metabolites were significantly changed between the MA-P and MA-D groups. The heat map visualized and depicted the distinction among the top 50 differential fecal metabolites (**Figure 5A**). Specifically, 21 metabolites including *3a, 7a, 12b-trihydroxy-5b-cholanoic acid*, *17alpha, 21-dihydroxypregnenolone*, etc., were distinctly higher in the Y-P group as measured with the Y-D group but were not changed in the middle-aged animals either in proestrus or diestrus. These metabolites in the MA-P group were found with a marked reduction when compared with the Y-P group. Because the remaining metabolites did not exhibit differences between proestrus or diestrus either in the Y or MA animals, the data from proestrus and diestrus were pooled for the Y and MA, respectively, to assess the age difference. A total of 14 metabolites were identified to be significantly higher in the MA group, including *18-acetoxy-1alpha*, *25-dihydroxyvitamin D3*, *3α, 12α-dihydroxy-5β-chola-7*, *14-dien-24-oic acid*, and *glycerol tributanoate.* Moreover, 15 metabolites were observed to be significantly lower in the MA group, namely, *montanol*, *DG (11M5/11D3/0:0)*, *MG (18:3 (9Z, 12Z, 15Z)/0:0/0:0)*, etc. (**Figure 5A**).

With KEGG annotation, we discovered 20 differentially enriched functional pathways between groups, in which steroid hormone biosynthesis, arachidonic acid metabolism, and primary bile acid biosynthesis were predominantly implicated (**Figure 5C**, *P* < 0.05).

### 3.5 Specific metabolites were associated with circulating hormonal indicators in menopausal transition

**Figure 6:**
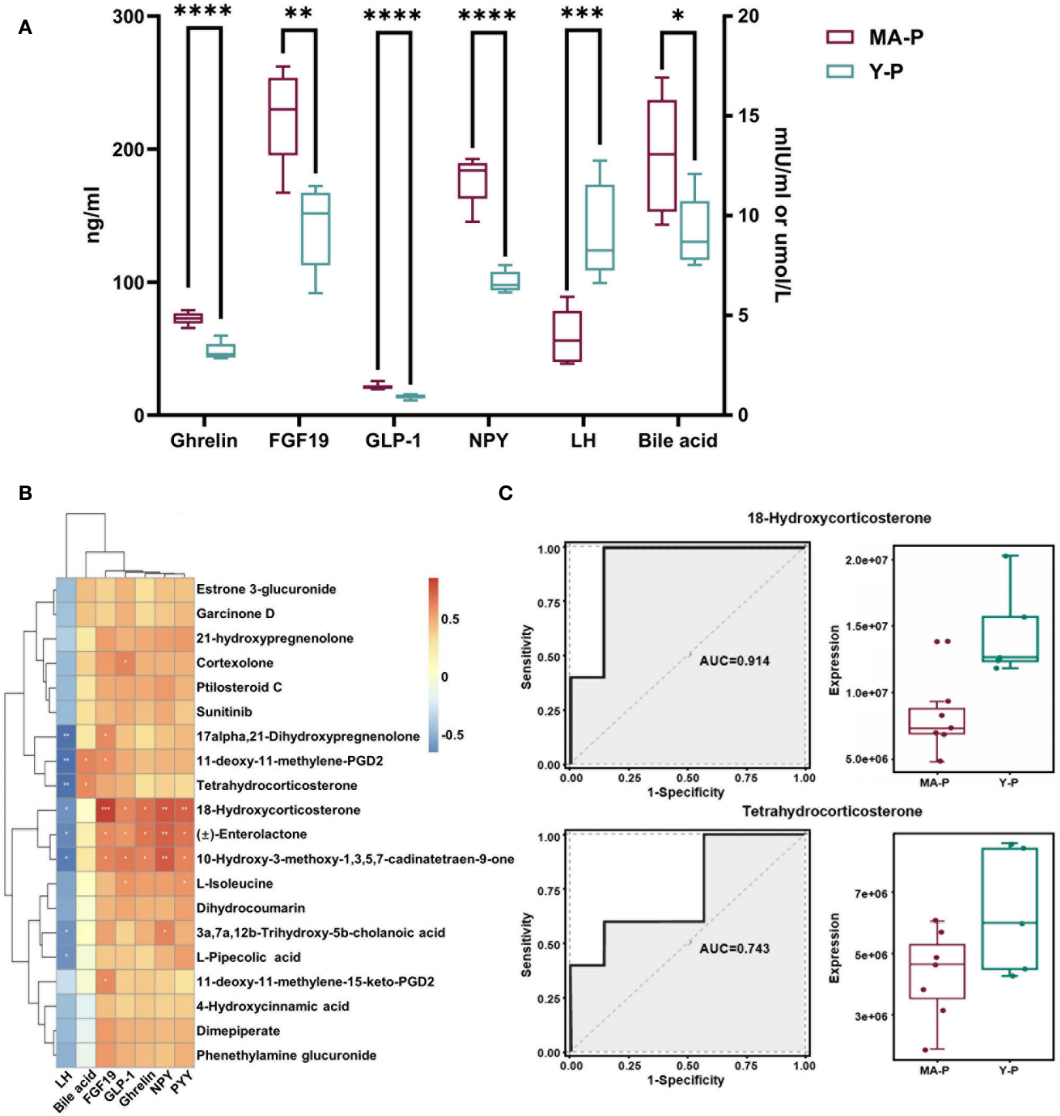
Associations among specific intestinal metabolites and circulating parameters in the menopausal transition. **(A)** The levels of luteinizing hormone (LH), bile acid, FGF19, and gut hormones including ghrelin, NPY, and GLP-1 in the serum of MA-P and Y-P rats (MA-P, *n* = 7; Y-P, *n* = 5). *P < 0.05, **P < 0.01; ***P < 0.001; ****P < 0.0001. **(B)** Heat map of correlations among LH, bile acid, and gut hormones *versus* specific intestinal metabolites. **(C)** Receiver operating characteristic analysis of two representative metabolites in the MA-P group compared with Y-P.

Menopausal transition is characterized by a reduced circulating LH surge (23–26, 46, 47), so we subsequently selected the metabolites lacking in hormone-associated changes (see **Figure 5A**) and estimated their associations with endocrine-related parameters in the blood. The serum levels of LH, FGF19, bile acid, and several gut hormones including ghrelin, NPY, and GLP-1 were found to be significantly changed in the MA-P rats (**Figure 6A**, *P* < 0.05). The Pearson correlation analysis revealed that metabolites encompassing *tetrahydrocorticosterone*, *11-deoxy-11-methylene-PGD2*, *18-hydroxycorticosterone*, and *10-hydroxy-3-methoxy-1,3,5,7-cadinatetraen-9-one* were observed to show a negative correlation to the LH (**Figure 6B**). *Tetrahydrocorticosterone* and *11-deoxy-11-methylene-PGD2*, however, were found to be positively related with bile acid. Furthermore, *18-hydroxycorticosterone*, *Enterolactone*, and *10-hydroxy-3-methoxy-1,3,5,7-cadinatetraen-9-one* were identified to have higher abundances and to be significantly correlated to FGF19, ghrelin, NPY, PYY, and GLP-1 (**Figure 6B**). We further selected representative metabolites and conducted a receiver operating characteristic (ROC) curve analysis, in which *18-hydroxycorticosterone* (area under the curve (AUC), 0.914; *P* = 5.4512E-5) and *tetrahydrocorticosterone* (AUC, 0.743; *P* = 0.015449) showed reductions in the MA-P rats (**Figure 6C**).

### 3.6 Analysis of correlations between different microbes and metabolites

**Figure 7:**
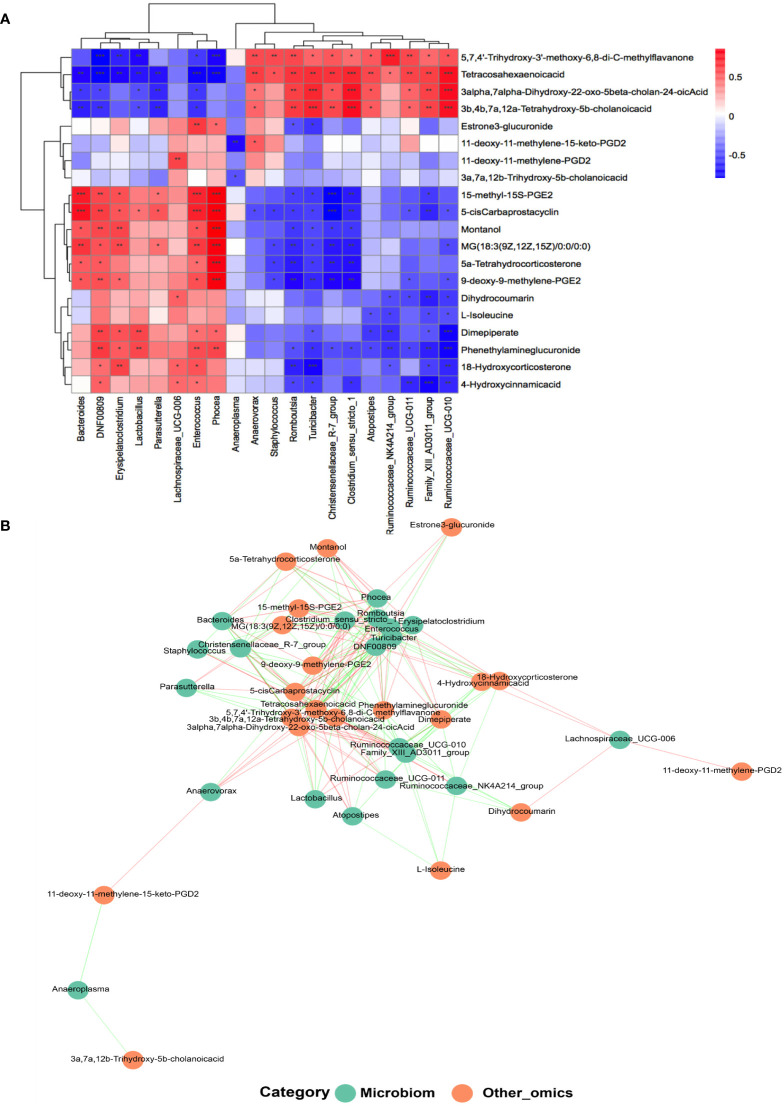
Analysis of the correlation between the gut microbiota and metabolites at the genus level. **(A)** Heat map of correlations between 20 kinds of metabolites and the gut microbiota at the genus level (*n* = 5–7 for each group). *P < 0.05, **P < 0.001; ***P < 0.0001. **(B)** Co-occurrence networks of the differential microorganisms and metabolites.

In the light of the hypothesis that fecal microbes contribute to the pathogenic progress of menopausal transition, microbe-related metabolic substances in different groups were recognized by performing Pearson’s correlation-based clustering analysis. At the genus level, the hormone-regulated metabolites that uniquely increased in the Y-P group (**Figure 5A**) showed varied correlations with different gut bacteria (**Figures 7A, B**). *Dimepiperate*, *dihydrocoumarin*, *18-hydroxycorticosterone*, *4-hydroxycinnamicacid*, and *phenethylamineglucuronide* showed a significant positive relevance with *DNF00809* and *Enterococcus* but a negative correlation with *Family_xIII_AD3011_group* and *Ruminococcaceae_UCG-011*, etc. *L-isoleucine* was negatively correlated with *Family_xIII_AD3011_group*, *Ruminococcaceae_UCG-010*, *Atopostipes*, and *Ruminococcaceae_ NK4A214_group*. The *3a, 7a, 12b-trihydroxy-5b-cholanoic acid* was negatively correlated with *Anaeroplasma*. The *11-deoxy-11-methylene-15-keto-PGD2* was positively correlated with *Anaerovorax*, while the 11-deoxy-11-*meth*ylene*-PGD2* was positively correlated with *Lachnospiraceae_UCG-006*.

The metabolites with age differences (**Figure 5A**) also showed differential correlations with the gut microbiota (**Figures 7A, B**). The *5,7,4’-trihydroxy-3’-methoxy-6,8-di-C-methylflavanone*, *tetracosahexaenoic acid*, *3alpha,7alpha-dihydroxy-22-oxo-5beta-cholan-24-oic acid*, and *3b,4b,7a,12a-tetrahydroxy -5b-cholanic acid*, which reduced in the Y but increased in the MA, were positively correlated with *Ruminococcaceae_UCG-010*, *Family_xIII_AD3011_group*, and *Ruminococcaceae_UCG-011* but negatively responded to *DNF00809*, *Enterococcus*, etc. However, *5-cis carbaprostacyclin*, *montanol*, and *MG (18:3 (9Z, 12Z, 15Z)/0:0/0:0)*, which increased in the Y but decreased in the MA, were negatively interrelated with *Romboutsia* and *Tunicibacter* but positively interrelated with *Phocea*, *Enterococcus*, etc.

### 3.7 Neuroendocrine aging traits in the MA rats

**Figure 8:**
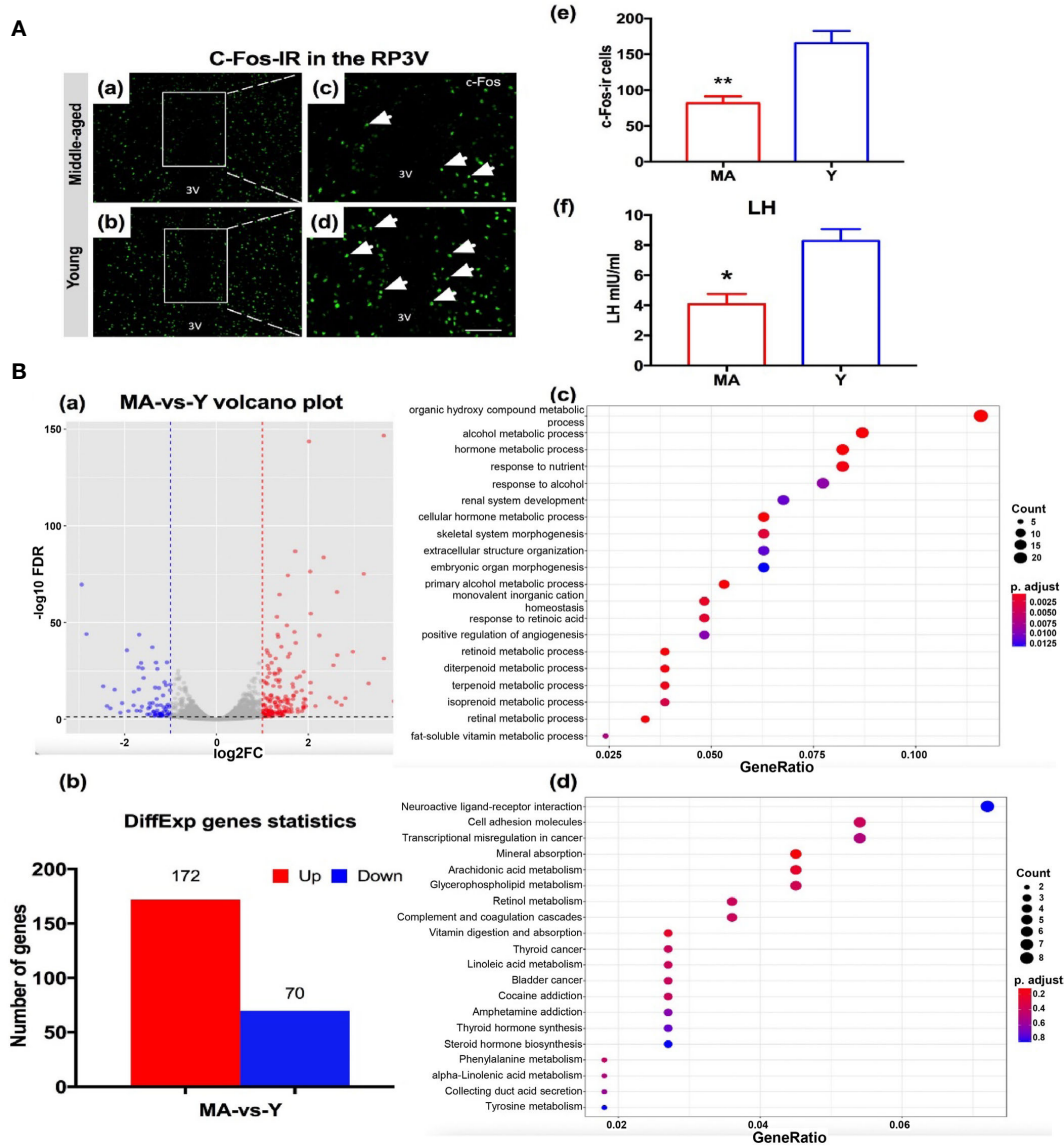
Neuroendocrine aging traits and hypothalamic transcriptome expression characteristics. **(A)** (a–d) Representative pictures showing c-Fos immunoreactive cells in RP3V of the middle-aged (MA) and young (Y) rats on the proestrus day. (e) Quantitative analysis of the c-Fos^+^ cells in the groups (*n* = 4, respectively). (f) Mean luteinizing hormone levels were measured using trunk blood collected from the MA and Y rats (*n* = 7, respectively). *P < 0.05, **P < 0.01. **(B)** (a) Volcano plot for differential gene expression analysis, (b) differentially expressed gene chart for the MA and Y rats, (c) GO enrichment analysis of biological processes of the differential genes, and (d) Kyoto Encyclopedia of Genes and Genomes pathway analysis. *n* = 3 for each group.

To detect the host hypothalamus response to a disturbed microbial community especially on the proestrus status, we evaluated the parameters including c-Fos expressions, transcriptomics of the anterior hypothalamus, and LH surge levels. The expression of c-Fos immunoreactive cells in the RP3V of MA group was significantly decreased by ~52% (**Figure 8A**), associated with a marked reduction of the LH value (**Figure 8A**). There existed no difference in either E2 or P between the MA and Y groups (data not shown). A total of 243 DEGs were screened out between the two animal sets, encompassing 172 genes that expressed considerably more in the MA group and 70 that were less expressed (**Figure 8B**). We noticed that genes expressing on the rise in the MA group correlated with organic hydroxy compound metabolic process, hormone metabolic process, and response to nutrient (**Figure 8B**). The KEGG entailed dominant pathways as neuroactive ligand–receptor interaction, cell adhesion molecules, and arachidonic acid metabolism (**Figure 8B**).

## 4 Discussion

Microbial origins of signaling molecules including metabolites and neurotransmitter homologs have been verified to have close associations with the host brain, causally linked to neuronal dysfunction and activation of the hypothalamic–pituitary–adrenal (HPA) axis (48, 49). Our present study revealed that *Bacteroides*, *Lachnospiraceae_NK4A136_group*, and *Ruminococcaceae_UCG-014* are the most abundant genera in the rats’ intestines, occupying almost 40% of the total sequence, which coincides with a previous report (50, 51). Supported by the results extracted from the LEfSe analysis, the microbial structure was identified to be shifting parallel with the dynamic gonadal hormones that changed over the course of the estrous cycles in both the Y and MA rats. Notably, genera such as *DNF00809*, *Lachnospiraceae_UCG-006*, *Eubacteriaceae*, and *Phocea* were enriched in the Y-P group when the preovulatory hormone was at a high level. The predominance of *Bacteroides*, *Lactobacillus*, and *Erysipelatoclostridium* was identified in the Y-D rats. Although the specific roles of these bacteria in estrous cycles remain to be determined, the altered bacterial genes were annotated with high activity in several pathways like G protein-coupled receptors and inositol phosphate metabolism. We speculate that these bacteria may serve as hinge influencing sex steroid hormones and shaping estrous cycles. In contrast, the genera composition in the MA female individuals, including *Clostridia*, *Anaerovorax*, *Rikenella*, *Tyzzerella_3*, *Carnobbacteriaceae*, and *Atopostipes* that was predominantly enriched in the MA-P and *Peptostreptococcaceae*, *Romboutsia*, and *Turicibacter* that abounded in the MA-D, were partly similar to the bacteria categories identified in intestinal inflammation and aging (52–57). These gut genera showed a high activity in phosphonate and phosphinate metabolism, amoebiasis, atrazine degradation, and chlorocyclohexane and chlorobenzene degradation. Seeing that the chronic systemic inflammation that produced the gut microbiota and its metabolites would change the brain–blood barrier (BBB) permeability and cause the pathophysiology of neuroinflammation and brain aging (52, 58), while fecal microbiota transplantation between the young and aged mice exacerbated aging-related inflammation in the central nervous system of the young mice, that of the aged mice was reversed and benefited from the young microbial profile (59). Moreover, the altered levels of gut microbiota treated by dietary amino acid would also be capable of modulating inflammation (60). Hence, we propose that the distinct inflammation-related gut microbiota and functional categories in the MA female individuals may eventually blunt the hypothalamus responsiveness to the systemic hormone feedbacks and propel the occurrence of neuroendocrine aging. Based on the recent study discovering that the depression in premenopausal women was potentially caused by an estradiol-degrading bacteria in the gut (61), though we did not see a fall in the circulating level of estradiol in the female rats experiencing menopausal transition, further functional studies are needed to clarify the causal relationship between the differential microbiota with its metabolites and female menopausal transition in our study. The current study implicates those alterations in the microbe–host interactions, and gut–brain communications are likely critical factors affecting the hypothalamic–pituitary–ovary (HPO) axis functionally during menopausal transition, though the circulating E2 and P levels associated with reproductive cyclicity have yet to be changed.

Altered neuronal activation and gene transcription in the RP3V of the hypothalamus during proestrus (62) are featured neuroendocrine phenotypes of menopausal transition, associated with a typical LH surge reduction and subfertility (47). Our RNA-sequencing data of anterior hypothalamus samples collected on proestrus day provided a sequence-based characterization of transcriptomics and validated the diversification of gene expressions between the MA and Y groups. Interestingly, the gut-associated metabolite analysis of this study revealed an age-related gut with active metabolism of lipids and lipid-like molecules, which was featured as another characteristic phenotype of the MA female individuals. This result implied that lipids and lipid-like molecules were largely consumed when female individuals transited into middle age. Particularly, 21 metabolites that experienced a hormone-related increase in the Y-P group lacked changes when the MA animals shifted from diestrus to proestrus—for instance, the concentration of *3a, 7a, 12b-trihydroxy-5b-cholanoic acid*, a primary bile acid molecule involved in carbohydrate digestion and absorption pathway, markedly dropped in the MA-P group, partly in accord with documented results (63, 64). Consisting of individual bile acid moieties, mucus, phospholipids, and biliverdin, the bile physiologically emulsifies fats, releases fat-soluble vitamins, and modulates cholesterol metabolism in the small intestines (65, 66). Specific bile acids differentially activate or suppress receptors such as farnesoid X receptor and the Takeda G protein-coupled receptor 5 (TGR5, also called as GPBAR1) as ligands (67). These receptors are expressed within a wide spectrum of organs spanning from the peripheral intestines and the central hypothalamus (68). In view of the fact that bile acids are made available to either inhibit or potentiate GABAA or N-methyl-D-aspartate (NMDA) receptors (69), the differential bile acids in the MA-P female individuals may potentially alter host gene expression patterns in the hypothalamus performing as systemic signaling molecules by way of circulating bile acids and gut hormones that pass through the BBB and elicit neuroendocrine changes of menopausal transition, that is, the downregulation of c-Fos in RP3V with concomitantly reduced LH levels. Moreover, *L-isoleucine* and *L-pipecolic acid* were identified to have the same characteristics as the bile acids in the MA rats. Considering that hypothalamic glutamate transporters/receptors and GABA receptors exhibited changes prior to irregular cycling (70), the missed hormone-related changes of *L-isoleucine* and *L-pipecolic acid* in the MA-P may also alter gene expression through inhibiting or potentiating GABAA or NMDA receptors. Therefore, we speculate that the gut microbiota-derived bile acids and amino acids may be associated with adjustments in glutamate and GABA signaling during menopausal transition through sensitizing the brain to counter environmental or hormonal insult and manipulating the risk and/or rate of neurological decline, as glutamate-mediated excitotoxicity is pertinent to neuronal activation and gonadotropin release. Consistent with the above-mentioned results, a GO enrichment analysis of the hypothalamus transcriptome indicated considerably altered genes in the MA group that were rich in the biological processes of organic hydroxy compound metabolic process, hormone metabolic process, and response to nutrient. The KEGG was mainly relevant to neuroactive ligand–receptor interaction, cell adhesion molecules, and arachidonic acid metabolism. Specific metabolites lacking in hormone response presented a high relevance to the circulating FGF19, bile acid, and the gut hormones including ghrelin, GLP-1, and NPY that were upregulated in the serum of the MA rats, which rendered a strong dependence on menopausal neuroendocrine phenotypes, indicating that those metabolites may directly serve as intermediaries for the shifting gut microbes to operate menopausal neuroendocrine traits or indirectly perturb gut hormones and bile acids and ultimately result in the typical reduced LH during menopausal transition. Nevertheless, whether alterations in the relevant concentrations of intestinal microbe-derived bile acids or the gut hormones are actually the physiological mapping of that in the central nervous system deserves further investigations (71, 72).

**Figure 9:**
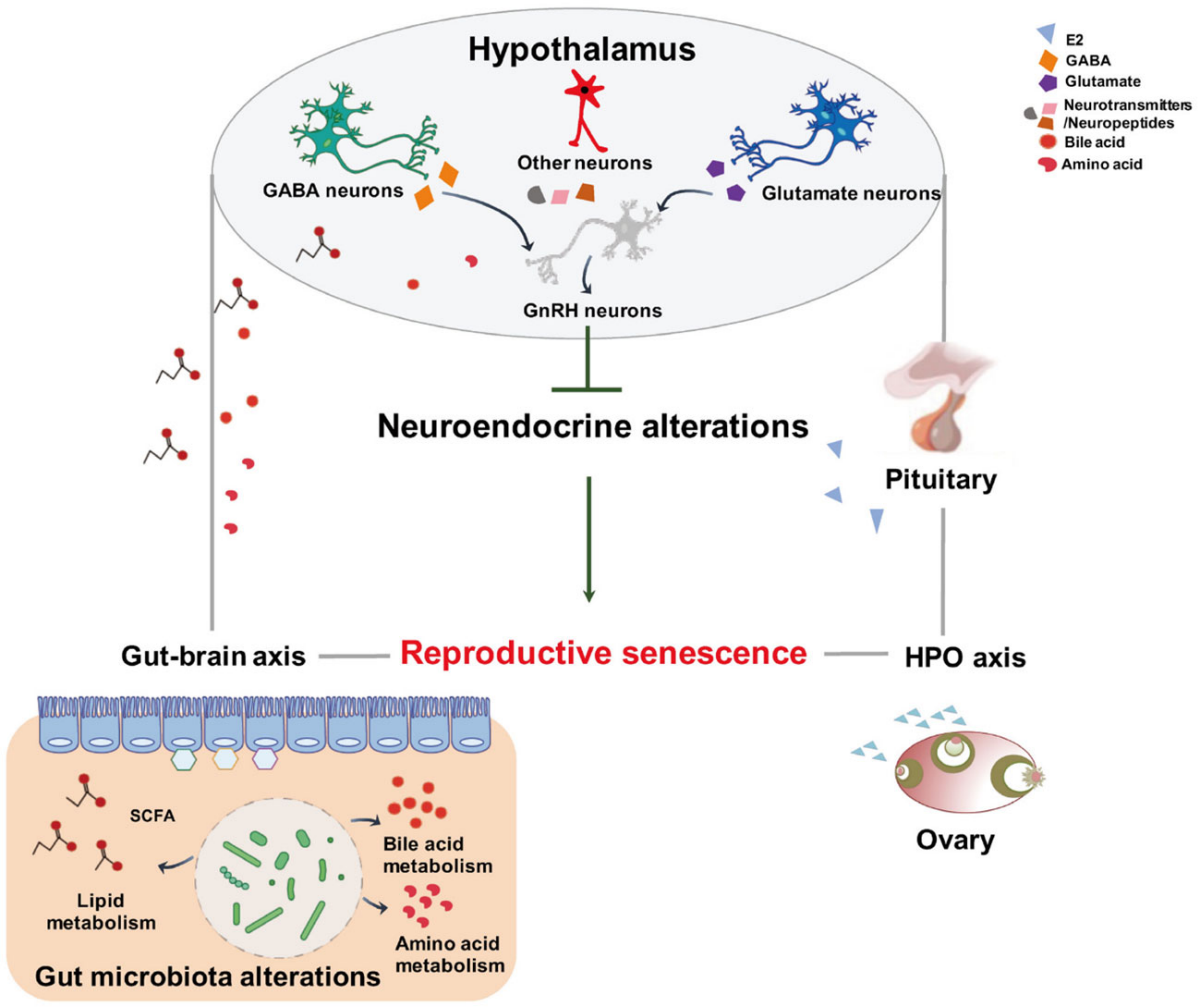
Schematic representation of the proposed mechanism of gut microbiota and metabolites modifying neuroendocrine aging to control female reproductive senescence. This diagram shows that altered intestinal bacteria produce metabolites that exert effects on the hypothalamus through the gut–brain axis. The gut origin molecules may blunt the responsiveness of the hypothalamus to systemic hormone feedback, resulting in reproductive senescence.

In summary, we herein reported for the first time that, during menopausal transition, the altered composition of the intestinal microflora at both proestrus and diestrus was accompanied by the variations of metabolomic changes in the feces. Importantly, the correlativity between the abundances of intestinal microbiome and the composition of metabolites denoted that the gut microflora-induced increase of lipid-producing metabolomics at the proestrus might downregulate gene expressions and neuronal activities in the hypothalamus and causally lead to the blunt surge of GnRH-LH. The findings suggest that phenotypical endocrine alterations of both the hypothalamus and the gut represent a critical period previous to the appearance of symptoms of reproductive senescence (**Figure 9**). Further expeditions are required to determine the serum metabolomics and to appraise the functional and structural effects on the brain with alterations in these microbe–host interactions and the resultant modifications in gut–brain communications.

From a translational perspective, these data signify that the transformation of the gut microbiomes and metabolomes emerges previous to the onset of the perimenopausal phenotype like irregular cycling. Managements to sustain the gut endocrine mechanisms, particularly the fatty acid-produced bacteria, could be a strategy to maintain endocrinological and neurological function in women (73–75).

## 5 Conclusions

Differential abundances and compositions of gut microflora and metabolites in mid-life were accompanied by alterations of neuronal activation and gene transcription in the RP3V of the hypothalamus, especially lipids and lipid-like molecules that were associated with an age-related gut with active metabolism. The fecal metabolites presenting close relationships with the typical reduced LH surge and elevated levels of bile acid, FGF19, and gut hormones may serve as mediations of the gut–brain dysfunction in neuroendocrine aging. This study extends our understanding of the putative causality of the gut microbial community with female reproductive senescence.

## Data availability statement

RNA sequencing data presented in this study are deposited in the SRA repository, accession number PRJNA1027788. Non-targeted LC-MS data presented in this study are deposited in the Metabolomics Workbench repository (76), Study ID ST003003. The data can be accessed directly via its Project DOI: http://dx.doi.org/10.21288/M8R41G.

## Ethics statement

The animal study was approved by the Institutional Animal Care and Use Committee of Fudan University. The study was conducted in accordance with the local legislation and institutional requirements.

## Author contributions

RD: Writing – original draft, Writing – review & editing, Formal Analysis, Investigation, Validation, Visualization. JH: Writing – original draft, Writing – review & editing, Formal Analysis, Investigation, Validation, Visualization. LC: Writing – review & editing, Investigation, Validation. RS: Writing – review & editing, Investigation, Validation. XQ: Writing – review & editing, Investigation, Validation. YW: Writing – review & editing, Investigation, Validation. YS: Conceptualization, Formal Analysis, Funding acquisition, Investigation, Methodology, Project administration, Supervision, Validation, Visualization, Writing – original draft, Writing – review & editing.
