# Supplementary material for: Gut microbiota and metabolites in estrus cycle and their changes in a menopausal transition rat model with typical neuroendocrine aging
Source: Front Endocrinol (Lausanne). 2023 Dec 15;14:1282694. doi: 10.3389/fendo.2023.1282694 (PMC10755682; doi:10.3389/fendo.2023.1282694)
Supplement: Supplementary file 1 [file Image_1.pdf]

A

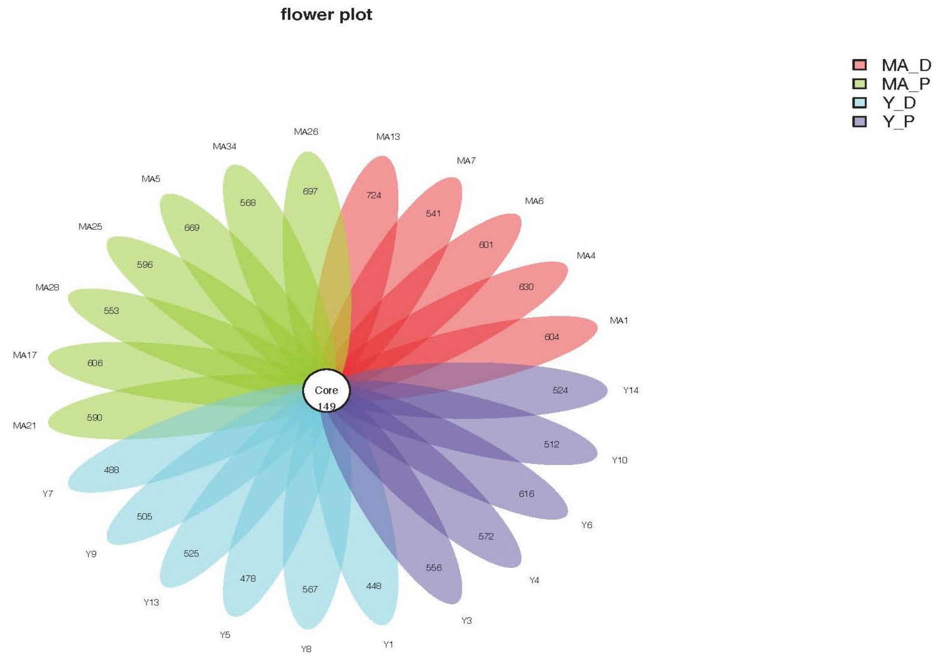


B

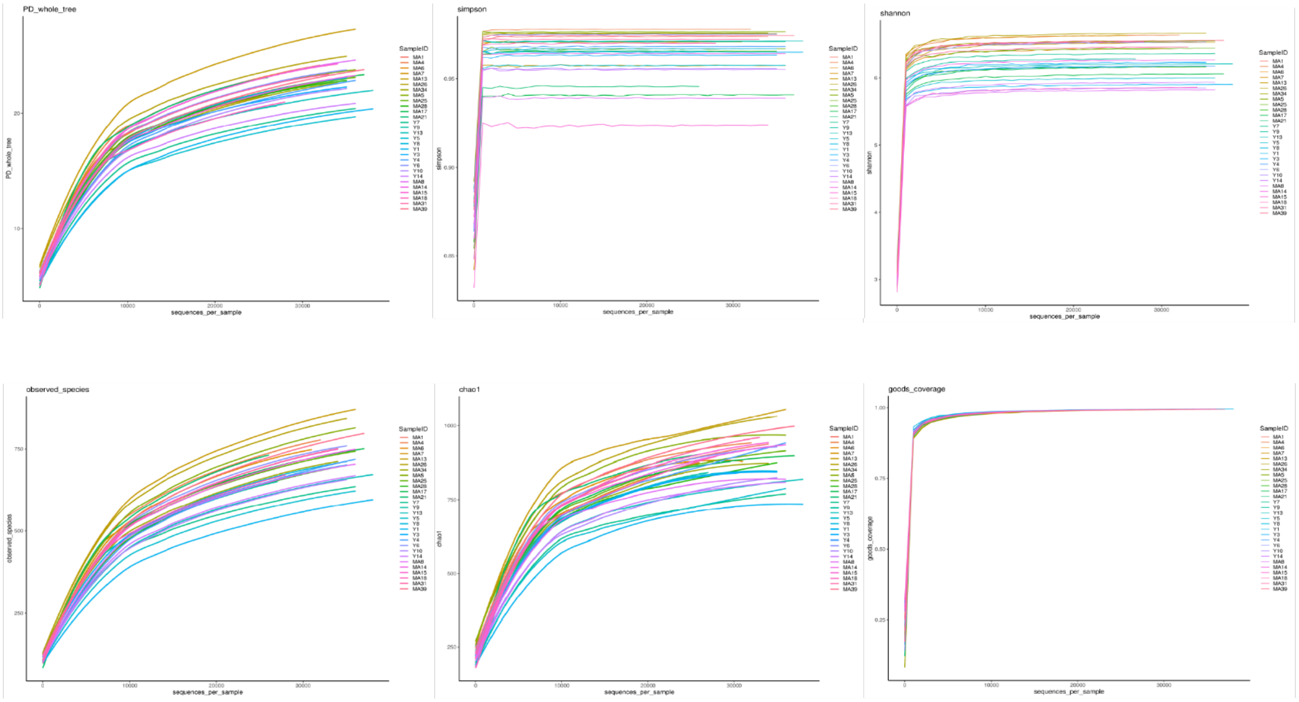


**Supplementary Figure S1. Sequencing data of 16S rRNA of middle-aged and young female rats in diestrus and proestrus. (A)** OTUs of each group. **(B)** Rare fraction curves show alpha diversities of each sample.

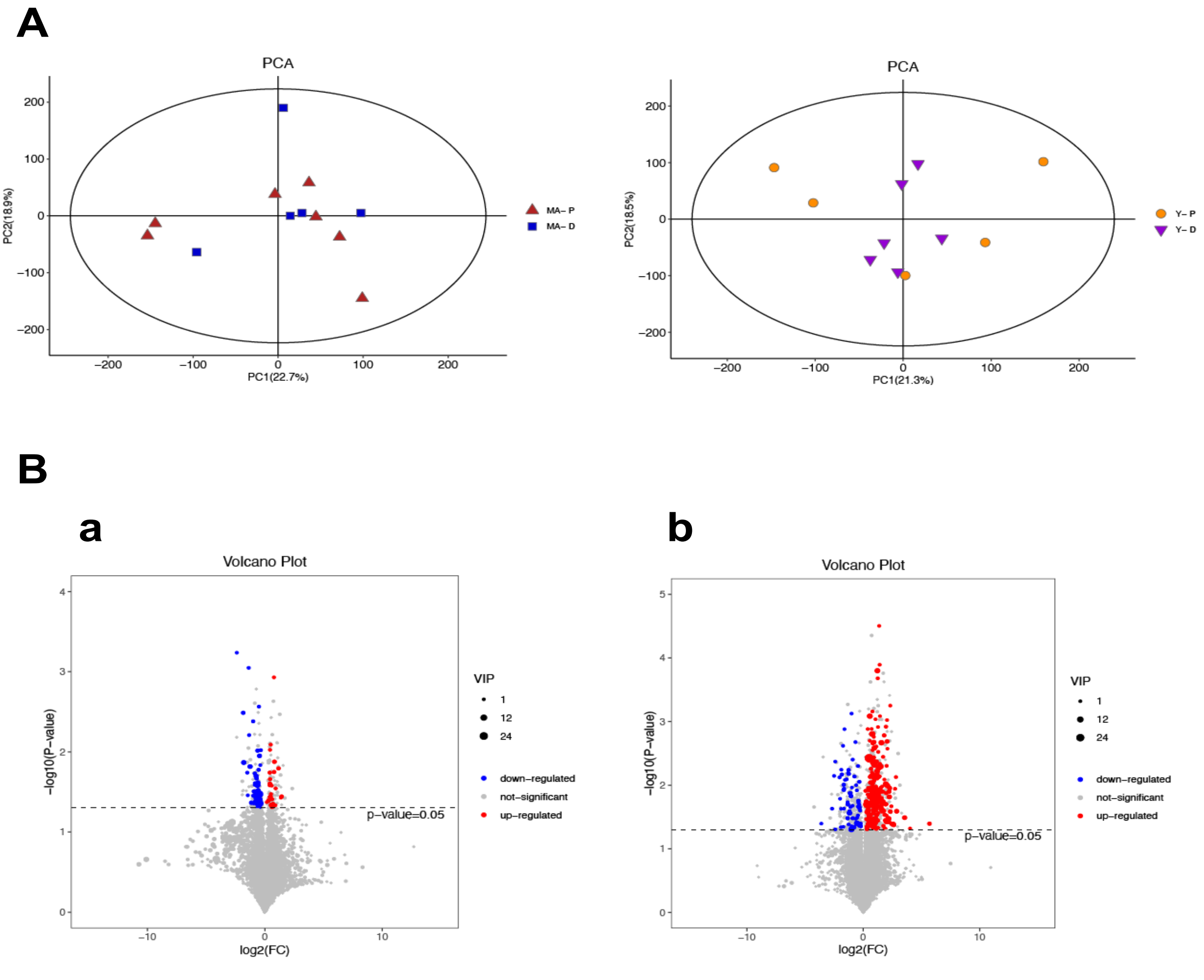


**Supplementary Figure S2. Differential metabolites of middle-aged and young female rats in diestrus and proestrus were screened out. (A)** PCA analysis shows beta diversity of metabolites between every two groups. **(B)** Volcano plots present up- and down-regulated metabolites of middle-aged rats **(a)** and young rats **(b)**.

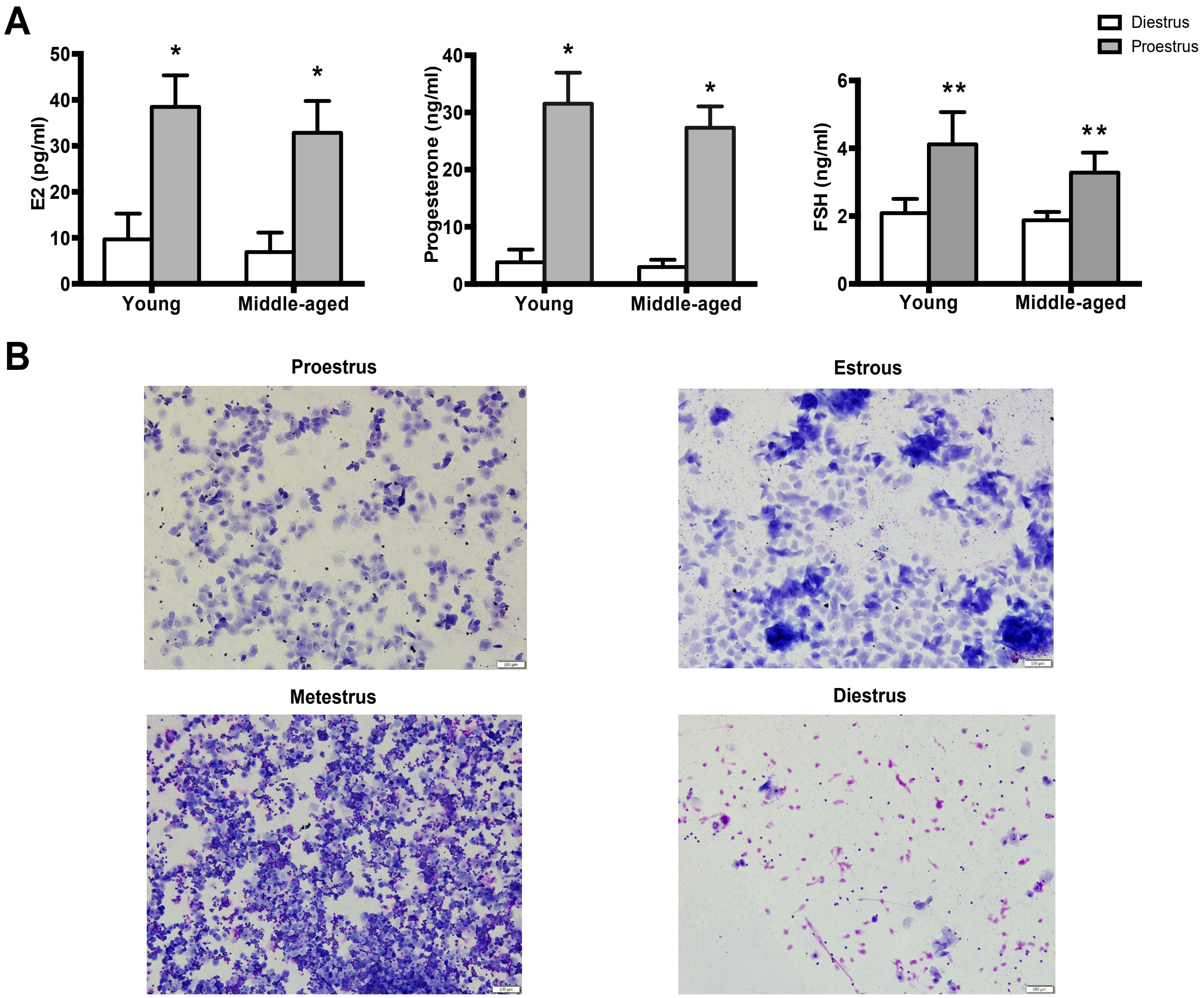


**Supplementary Figure S3. Reproductive phenotypes of rats. (A)** The serum levels of E2, Progesterone and FSH between the young and middle-aged rats at either diestrus or proestrus stage were listed by bar charts (n = 7, respectively). *$P < 0.05$, **$P < 0.01$. **(B)** The representative cytologies of the four estrous stage were presented.

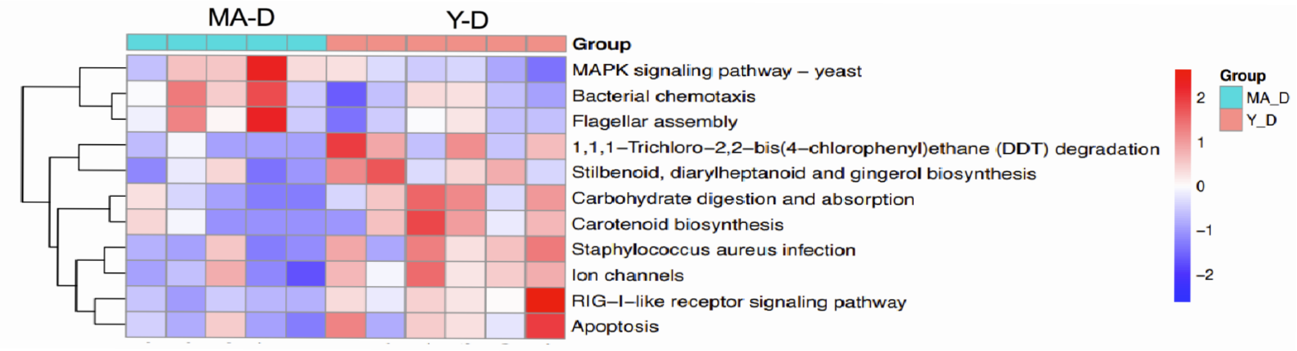


**Supplementary Figure S4.** Differentially enriched KEGG pathways of gut microbes between the middle-aged and the young rats at diestrus stage. (MA-D, n = 5; Y-D, n = 6)
